# Geriatrisches Assessment in der Zahnmedizin: Eine Übersicht zu Kaufunktionstests

**DOI:** 10.1007/s00391-023-02208-w

**Published:** 2023-06-26

**Authors:** Maximiliane Amelie Schlenz, Alexander Schmidt, Clara Sophie Gäbler, Gerald Kolb, Bernd Wöstmann

**Affiliations:** 1 Zentrum für ZMK-Heilkunde – Poliklinik für Zahnärztliche Prothetik, Justus-Liebig-Universität Gießen, Schlangenzahl 14, 35392 Gießen, Deutschland; 2 Ambulanz für Physikalische und Rehabilitative Medizin, Spezialisierte Geriatrische Diagnostik und Therapie, „Ärztehaus“ am Bonifatius Hospital Lingen, Lingen, Deutschland

**Keywords:** Geriatrische Mundgesundheit, Alterszahnmedizin, Mundgesundheitsdienste, Kaufunktion, Patientenversorgung, Geriatric oral health, Geriatric dentistry, Oral health care services, Mastication, Patient care

## Abstract

Durch die steigende Lebenserwartung und den damit verbundenen demografischen Wandel sind immer mehr Menschen auf Pflege angewiesen. Um erste Hinweise auf einen möglichen notwendigen zahnärztlichen Behandlungsbedarf zu geben, haben sich im zahnmedizinischen Bereich Kaufunktionstests als Assessmentinstrumente bewährt. Im vorliegenden Beitrag wird dem Leser eine Übersicht über vorhandene Kaufunktionstests und deren Durchführung gegeben. An dieser Stelle ist darauf hinzuweisen, dass ein Patient mit Schmerzen unabhängig von einem Kaufunktionstest unverzüglich einer Zahnärztin/einem Zahnarzt vorgestellt werden sollte. Weiterhin ersetzen Kaufunktionstests keine zahnärztlichen Routineuntersuchungen, könnten jedoch auch (zahn-)medizinischen Laien Hinweise darauf geben, ob ein Termin in einer Zahnarztpraxis vereinbart werden sollte oder ein zahnärztliches Konsil notwendig ist.

## Abstract (en)

Due to increasing life expectancy and the associated demographic changes, more and more people are dependent on care. To identify a possible need for dental treatment, chewing function tests as assessment instruments have proven their effectiveness. In this article, the reader is given an overview of existing chewing function tests and their implementation. It is important that a patient with pain should be presented to a dentist immediately, regardless of whether a chewing function test is performed. Furthermore, chewing function tests are not a substitute for routine dental examinations, but they could provide information to (dental) laypersons as to whether an appointment should be arranged in a dental practice or whether a dental consultation is necessary.

Innerhalb der geriatrischen Pflege wurden Assessmentinstrumente etabliert, die eine schnelle und einfache Diagnostik erlauben. Um auch in der Zahnmedizin behandlungsbedürftige Patienten zielgerichtet zu identifizieren, wurden verschiedene Kaufunktionstests entwickelt, welche die Zerkleinerung, Durchmischung oder Kauleistung mithilfe von Testnahrung untersuchen. Diese sollten auch von nichtzahnmedizinischem Pflegepersonal und von Angehörigen anzuwenden sein, denn häufig sind Patienten – trotz objektiv schlechter Mundgesundheit – subjektiv mit ihrem Zahnersatz zufrieden. Dies kann zu einer Mangel- oder Fehlernährung führen, sodass der Mundgesundheitszustand unmittelbar im Zusammenhang mit der Allgemeingesundheit steht.

Der Anstieg der Lebenserwartung und der damit verbundene demografische Wandel führen in einer Vielzahl an Ländern zu einer kontinuierlichen Zunahme der älteren Bevölkerung. Dabei nimmt gleichsam die Anzahl derjenigen Menschen zu, welche auf häusliche Pflege bzw. Hilfe in Pflegeheimen angewiesen sind [18]. Die zahnärztliche Versorgung sowie die Aufrechterhaltung einer guten Mundhygiene stehen jedoch leider oftmals nicht im direkten Fokus der Pflege, was durch den Mangel an Pflegekräften in Verbindung mit geringer zur Verfügung stehender Zeit, der Zunahme von Allgemeinerkrankungen bis hin zur Multimorbidität der betroffenen Patienten sowie steigenden Kosten des Pflegeaufwands verstärkt wird [25]. Erschwerend kommt hinzu, dass es für nichtzahnärztliches Personal teilweise sehr schwer ist, den Zahnstatus der Patienten zu beurteilen. Darüber hinaus ist es zahlreichen Bewohnern in Pflegeheimen, klinischen Einrichtungen oder in häuslicher Pflege nicht mehr möglich, selbstständig einen Termin zu vereinbaren und sich in einer Zahnarztpraxis vorzustellen. Aus diesem Grund wird der Mundgesundheitsstatus nur selten überwacht. Dies konnte innerhalb der Fünften Deutschen Mundgesundheitsstudie bestätigt werden [11]. Darin erfolgte eine Unterteilung der Altersgruppe der älteren Senioren zwischen 75 und 100 Jahren in Patienten mit und ohne Pflegebedarf. Innerhalb der Gruppe ohne Pflegebedarf hatten insgesamt 73,9 % der Befragten innerhalb der letzten 12 Monate eine zahnärztliche Kontrolluntersuchung oder Behandlung erhalten; im Vergleich dazu waren es in der Gruppe mit Pflegebedarf nur 52,7 %. Weitere Studien beschreiben mit 15 % [4], 33,3 % [11] und 45,5 % [33] sogar einen noch geringen Anteil von Pflegeheimbewohnern, die eine regelmäßige, zahnärztliche Kontrolluntersuchung erhalten.

Auf der Seite der Patienten kommt das „Paradoxon des Alterns“ hinzu [9]. Deutlich wird dies am Beispiel des Zahnersatzes, an welchen Patienten mit zunehmendem Alter häufig nur geringe Erwartungen haben. Dies kann zu einem „Underreporting“ führen, sodass Probleme mit bestehendem Zahnersatz oft zu spät oder gar nicht bemerkt und behandelt werden [34]. In einer vorangegangenen Untersuchung konnte zudem festgestellt werden, dass ein als unzureichend bewerteter Zahnersatz mit einer tendenziell schlechteren Kauleistung korreliert, was insbesondere für ältere Menschen zutrifft [2] und in einer Mangel- oder Fehlernährung resultieren kann [3, 14, 15, 19, 24, 36–38]. Bei Patienten, welche noch eigene Zähne haben, ergibt sich die Gefahr der Entstehung weiterer kariöser Läsionen, wobei gerade bei Älteren die Wurzelkaries ein erhebliches Problem darstellt. Auch Parodontalerkrankungen und die Entstehung von Mundschleimhautveränderungen bis hin zu Tumorerkrankungen werden begünstigt und bleiben häufig unentdeckt [39].

Aus diesem Grund besteht ein dringender Bedarf an einfachen und zuverlässigen Methoden für Pflegepersonal und Angehörige, die Patienten zu identifizieren, welche subjektiv mit ihrer Mundsituation zufrieden sind, objektiv aber einen hohen Bedarf an zahnärztlicher Behandlung haben, um sie dann möglichst zielgerichtet einer zahnärztlichen Behandlung zuzuführen.

## Pflegeassessments

Zur einfachen Detektion sowie standardisierten Sammlung von medizinischen Informationen wurden Assessmentinstrumente innerhalb der Pflege etabliert. Im Bereich der Zahnmedizin eignen sich v. a. Kaueffizienztests als Assessmentinstrumente, um die Mundgesundheit von Patienten in Bezug auf die Fähigkeit Nahrung zu zerkleinern oder zu durchmischen zu beurteilen [15, 26].

Als einflussnehmende Faktoren für die Kaueffizienz gilt in erster Linie die vorhandene Anzahl an Zahnpaaren und deren Kontakt zueinander (Okklusion). Je geringer die Anzahl der Zahnpaare, desto geringer die Kaueffizienz. Dabei bedingen die Seitenzähne einen Großteil der Kaueffizienz [6, 12, 16, 22]. Bei einer Anzahl von weniger als 8 okkludierenden Zahnpaaren gilt die Kaueffizienz als reduziert [13]. Zudem können Erkrankungen wie Parodontitis, Karies oder Zahnlockerungen die Kaueffizienz verringern [22].

Neben den natürlichen Zähnen haben auch das Vorhandensein und die Art von Zahnersatz einen Einfluss auf die Kaueffizienz, dabei kann insbesondere herausnehmbarer Zahnersatz grundsätzlich die Kaueffizienz eines natürlich vollbezahnten Patienten nicht ersetzen [6]. Während implantatgetragener Zahnersatz noch die höchste Kaueffizienz hervorbringt, limitiert bei Teilprothesen die unter der Auflagefläche liegende Schleimhaut in Verbindung mit der fehlenden direkten Krafteinleitung in den Kieferknochen durch Zähne oder Implantate die Kaukraft. Die geringste Kaueffizienz besitzen daher Patienten mit Totalprothesen, da diese vollständig schleimhautgelagert sind [13, 21, 22, 40].

Auch die Ausführung und der Zustand des Zahnersatzes haben einen wesentlichen Einfluss auf die Kaueffizienz. Diese verringert sich, wenn die Passfähigkeit und Retention der Halteelemente abnehmen. Dieser Prozess hat einen negativeren Einfluss als der Verlust eines okkludierenden Zahnpaares [13].

## Ermittlung der Kaueffizienz

Die Verfahren zur objektiven Ermittlung der Kaueffizienz lassen sich grundsätzlich auf Basis von Zerkleinerungs‑, Durchmischungs- sowie Kauleistungsuntersuchungen von Testnahrung einteilen. Bei subjektiven Verfahren erfolgt die Einschätzung durch Fragebogen oder Interviews, welche eine hohe patientenspezifische Komponente beinhalten [7]. Daher soll im Folgenden nur auf die objektiven Testverfahren näher eingegangen werden.

Bereits im Jahr 1950 nutzten Manly und Braley [17] die Siebmethode zur Bestimmung der Kaueffizienz von zuvor zerkauter Testnahrung, welche nach wie vor als „Goldstandard“ gilt. Allerdings ist der Zeitaufwand relativ hoch, und das notwendige Vorhandensein von Laborgeräten sowie Sieben und Rüttlern erschwert die Durchführung [20, 32]. So wurden zur einfacheren Umsetzung später Studien mit optischer Auswertung der Partikel durchgeführt. Hierbei sind jedoch ebenfalls technische Geräte sowie die Anschaffung von Bildbearbeitungsprogrammen notwendig [5].

Als typische Testnahrungsmittel dienen Nüsse, Brot, Fisch, Fleisch oder Gemüse. Zur bestmöglichen Zerkleinerung sollte das Testnahrungsmittel jedoch ein gutes Bruchverhalten aufweisen, da der Speisebolus nicht verklumpen oder sich auflösen darf. Weiterhin sollte es unabhängig von der Art des Zahnersatzes bzw. der Anzahl okkludierender Zahnpaare zerkleinert werden können [20]. Diesen Ansprüchen werden beispielsweise rohe Karotten gerecht; sie weisen zu Beginn eine weitgehend homogene und vergleichbare Konsistenz auf, lassen sich einfach in eine geeignete Form schneiden, liegen nach dem Zerkleinern in Bruchteilen vor und verklumpen nicht. Zudem sind sie kostengünstig und vielen Patienten als Nahrungsmittel bekannt, wodurch ein realitätsnahes Kaumuster erzielt werden kann [1].

Neben natürlicher Testnahrung kann künstliche Testnahrung wie z. B. Silikonwürfel, gehärtete Gelatine oder Fruchtgummi verwendet werden. Dabei sollte jedoch darauf geachtet werden, dass Testmaterial entsprechend der Leistungsfähigkeit der Patienten in Bezug auf die Kaumuskulatur, Kaukraft bzw. Bezahnung auszuwählen, da zu harte Materialien u. U. nicht zerkaut werden können [6, 8, 17].

Anstelle der Zerkleinerungsverfahren können Durchmischungsverfahren – mit Kaugummis oder Paraffinwachs als Testnahrung – zur Messung der Kaueffizienz verwendet werden, welche vergleichbare Ergebnisse wie Zerkleinerungsverfahren zeigen [28]. Vor allem für Patienten mit Schluckstörungen oder bei Patienten nach einem Schlaganfall werden Durchmischungstests im Vergleich zu Zerkleinerungstestverfahren empfohlen, da bei Letzteren eine geringere Aspirationsgefahr besteht [7, 31]. Allerdings stehen bei Kaugummis die Farbdurchmischungen im direkten Zusammenhang mit den Materialeigenschaften. An dieser Stelle erschweren rheologische Faktoren und die damit verbundene Änderung der Härte mit zunehmendem Kauvorgang die Vergleichbarkeit unterschiedlicher Durchmischungstests [7].

Neben den beschriebenen Testprinzipien sind noch weitere Verfahren bekannt, bei welchen nach einer definierten Anzahl von Kauzyklen von Fruchtgummi der Glucosegehalt oder die Farbänderung des Speichels gemessen wird. Ähnlich wird nach dem Kauen auf einer Kapsel mit enthaltenen Farbstoffkugeln die Änderung durch Spektralphotometer ausgewertet [7, 10, 23]. Dabei ist zu bedenken, dass sich das Kauen auf einer Kapsel nur schwer mit einem natürlichen Kauvorgang vergleichen lässt. Wenngleich diese beiden Formen der Kaufunktionstests innerhalb von Studien beschrieben wurden, finden diese im Alltag nur selten Anwendung.

## Mögliche Assessmentinstrumente zur Ermittlung der Kaueffizienz

Im Folgenden wird eine Übersicht über vorhandene Assessmentinstrumenten und deren praktische Umsetzung am Patienten dargestellt. Eine tabellarische Übersicht beinhaltet Tab. 1.

**Tab. 1:** Übersicht der verfügbaren Kaufunktionstests

Kaufunktionstest	Prinzip	Zeitaufwand	Kaugut	Allgemeine Verfügbarkeit	Handlungsempfehlungen	Digitale Form vorhanden
	(D) Durchmischung (Z) Zerkleinerung (K) Kauleistung	(V) Vorbereitung (D) Durchführung (A) Auswertung		(KG) Kaugut (GS) Geräte/Software		
*Kaufunktionstest nach Schimmel *[29]	D	V: gering D: gering A: mittel	Spezielle Kaugummis	KG: spezielle Kaugummis nur für Forschungszwecke von der Universität Bern erhältlich GS: Auswertungssoftware frei im Internet verfügbar	Indirekt	Ja
*Kaufunktionstest nach Sato et al. *[27]	D	V: hoch D: gering A: mittel	Testwürfel aus Paraffinwachs	KG: eigene Herstellung der Testwürfel erforderlich GS: digitale Bildauswertungssoftware erforderlich	Indirekt – 3 Gruppen (gut, mittel und schlecht) werden vorgeschlagen	Nein
*Kaufunktionstest nach Nakasima et al. *[23]	K	V: hoch D: gering A: hoch	Spezielle Kapsel mit einem Testgranulat	KG: eigene Herstellung der Testkapsel erforderlich GS: Spektralphotometer	Indirekt	Nein
*Kaufunktionstest nach Slavicek *[35]	Z/K	V: gering D: hoch A: mittel	Spezielle Fruchtgummis	KG/GS: spezielle Fruchtgummis und Auswertungsgerät auf Webseite des Herstellers erhältlich	Indirekt	Ja
*Mini Dental Assessment *[39, 32]	Z	V: mittel D: gering A: gering	Karotten	KG: handelsübliche Karotten GS: analoge Papierform auf Webseite der Universität Gießen frei verfügbar; digitale Form derzeit nur für Forschungszwecke erhältlich	Direkt	Ja (jedoch derzeit nur für Forschungszwecke)
*Kaufunktionstest nach Huggare und Skindhöj *[10]	Z	V: hoch D: gering A: hoch	Synthetische Testgummis aus Carnaubawachs und Bariumsulfat	KG: eigene Herstellung der Testgummis erforderlich GS: Spektralphotometer	Indirekt	Nein

### Kaufunktionstest mit der Siebmethode

Die Siebmethode ist die älteste bekannte Methode der Kaufunktionstests, dazu werden dem Patienten beispielsweise Erdnüsse zum Zerkauen gegeben [17]. Diese soll der Patient mit einer definierten Anzahl an Kauzyklen zerkauen, im Anschluss wird der Bolus in Siebe verschiedener Maschenweiten von grob nach fein gegeben, mit Wasser abgespült und getrocknet. Danach werden die einzelnen Fraktionen aus den aufeinander folgenden Siebebenen gewogen. Zur Ermittlung des Ergebnisses werden unterschiedliche Auswertungsmethoden beschrieben – wie Verhältnis der Fraktionen, durchschnittliche Partikelgröße – sodass in der Regel ein kontinuierlicher Wert resultiert [30]. Das Verfahren wird bis heute gerade im Kontext vergleichender prospektiver Studien mit unterschiedlichem Kaugut, meistens jedoch Nüssen oder Mandeln, sehr oft verwendet. Die klinische Erfahrung mit der Siebmethode zeigt allerdings, dass gerade pflegebedürftige Patienten insbesondere mit Totalprothesen diese Testnahrung nicht akzeptieren [39, 40]. Zudem sind Unverträglichkeiten oder Allergien im Zusammenhang mit Nüssen im Vergleich mit anderem Kaugut häufig beschrieben. Ein Test-Set-up, aus dem eine direkte Handlungsempfehlung abgeleitet würde, ist den Verfassern nicht bekannt.

### Kaufunktionstest nach Schimmel

**Abb. 1:**
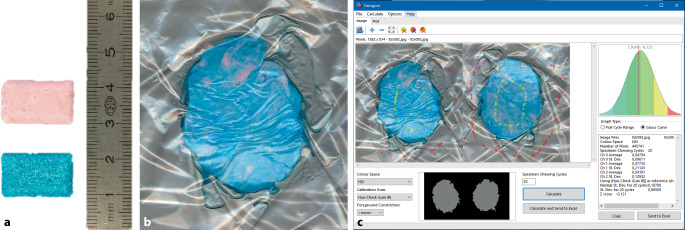
Kaufunktionstest nach Schimmel. **a** Zweifarbige Kaugummis (Hue-check-Gum) vor dem Kaufunktionstest. **b** Kaufunktionstest durchgeführt, Bolus für die Analyse vorbereitet. **c** Beispielhafte Darstellung der ViewGum®-Software

Bei dem Kaufunktionstest nach Schimmel [29] werden dem Patienten zwei unterschiedlich farbige Kaugummis (rosa/blau, Hue-Check Gum®, Klinik für Rekonstruktive Zahnmedizin und Gerodontologie, Bern, Schweiz, Abb. 1a) zum Zerkauen gegeben. Der Patient wird danach gebeten, die Kaugummis mit einer definierten Anzahl an Kauzyklen zu zerkauen und zu durchmischen. Nach Herausnahme wird die Durchmischung visuell beurteilt und in eine Gradskala eingeteilt. Dafür wird der Bolus auf eine Dicke von einem Millimeter gepresst (Abb. 1b), in abgeflachter Form gescannt und mit einem Bildbearbeitungsprogramm ausgewertet. Der Grad der Durchmischung wird durch das Verhältnis unvermischter sowie vermischter Anteile im Vergleich zur gesamten Oberfläche ausgewertet.

Zur Auswertung des Durchmischungsgrades wurde eine Software (ViewGum®software, dHAL Software, Griechenland) entwickelt, welche kostenlos heruntergeladen werden kann (www.dhal.com). Mithilfe des Programms werden die Farbe der Pixel des Kaugummis gemessen und die Standardabweichung der jeweiligen Farbtonkomponenten berechnet (Abb. 1c). Als Ergebnis zeigt sich, dass eine hohe Standardabweichung einen höheren Anteil an undurchmischten Kaugummianteilen und damit eine geringere Kaueffizienz darstellt. Eine unmittelbare Handlungsanweisung zeigt der Test nicht, vielmehr ergibt sich ein kontinuierlicher Wert für den Durchmischungsgrad.

### Kaufunktionstest nach Slavicek

**Abb. 2:**
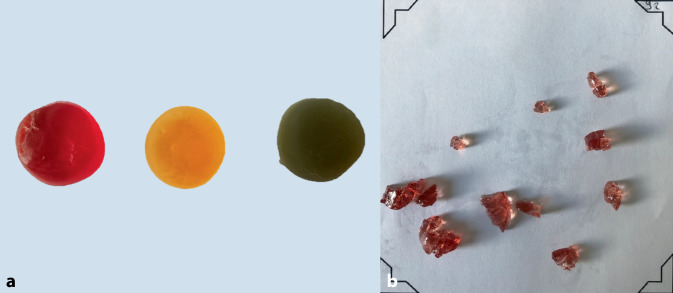
Kaufunktionstest nach Slavicek. **a** Fruchtgummis in unterschiedlichen Härtegraden (weich, medium, hart). **b** Ausgebreiteter Bolus nach dem Kaufunktionstest mit weichem Fruchtgummi

Beim Kaufunktionstest nach Slavicek [35] werden dem Patienten 3 Fruchtgummis mit jeweils unterschiedlichen Härtegraden (weich, mittel, hart; Abb. 2a) zum Zerkauen gegeben. Dabei wird jeder Härtegrad auf der linken, der rechten sowie auf beiden Seiten der Kieferhälften für jeweils 30 s zerkaut. Aus dem Grad der Zerkleinerung der unterschiedlichen Fruchtgummis unterschiedlicher Härtegrade kann hierbei ebenfalls eine Aussage über die Kauleistung getroffen werden. Nach dem Kauen werden die Fruchtgummis auf einem Auswertungsblatt glatt ausgestrichen (Abb. 2b). Das Blatt wird in einen speziellen Auswertungsautomaten (Orehab Minds, Stuttgart) gegeben und digital ausgewertet, wobei die Daten auf eine Online-Plattform hochgeladen werden. Nach der Analyse erhält sowohl der Untersucher als auch der Patient eine Auswertung. Diese ist in eine Wertetabelle mit Normbereichen sowie Grenzbereichen unterteilt. Weiterhin wird die Anzahl der Partikel pro Kausequenz, pro Kauseite sowie pro Härtegrad angegeben. Zudem wird eine verbale Beschreibung über die Kaufunktion im Vergleich zu hinterlegten Daten aufgezeigt.

### Mini Dental Assessment

**Abb. 3:**
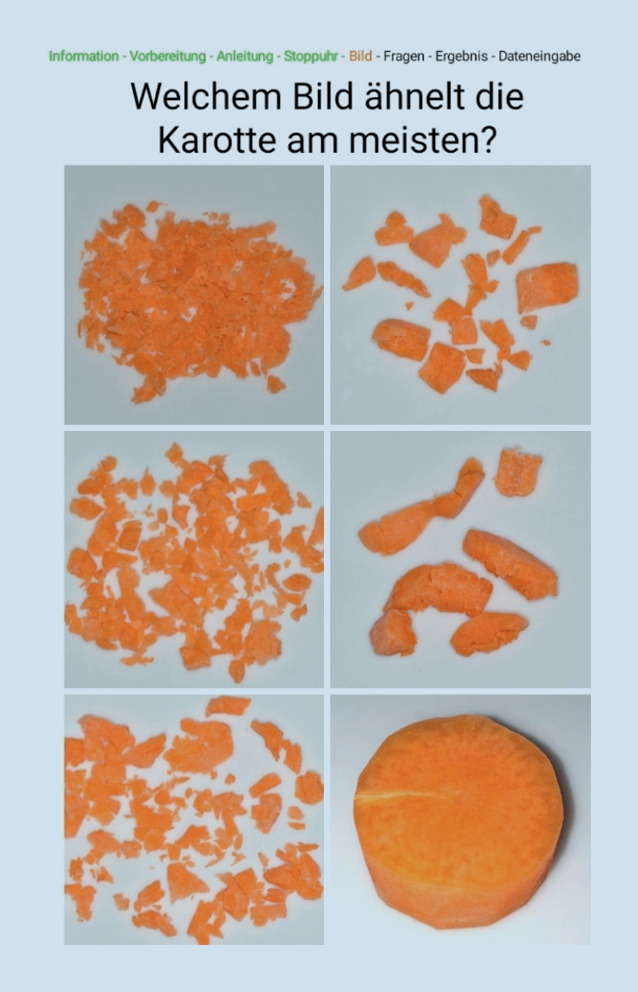
Bewertungsskala des Mini-Dental-Assessment für unterschiedliche Zerkleinerungsgrade der Karotte (Bildschirmfoto der MDA-App)

Einen einfachen Ansatz zur Detektion der Kaufunktion und des damit verbundenen Mundgesundheitszustandes bildete das Mini Dental Assessment (MDA) [39]. Dabei wird dem Patienten ein Stück Karotte definierter Größe (1 cm Höhe, 2 cm Durchmesser) für 45 s zum Kauen gegeben. Wie bei allen anderen Kaufunktionstest auch ist es dabei wichtig, dass der Patient keinen Anteil der Karotte herunterschluckt, damit im Anschluss eine visuelle Bewertung erfolgen kann. Nach dem Vergleich mit einer Bewertungsskala (Abb. 3) wird anhand der Zeit seit dem letzten Zahnarztbesuch sowie des Alters des letzten Zahnersatzes eine Punktzahl ermittelt. Als Ergebnis ergibt sich eine Handlungsempfehlung, ob ein Besuch in einer Zahnarztpraxis notwendig bzw. ratsam ist, oder ob eine regelmäßige Routinekontrolle ausreicht.

Neben der analogen Papierform (Auswertungsblatt steht unter www.ukgm.de/ugm_2/deu/ugi_zap/PDF/MDA_Formular.pdf zum Download zur Verfügung) wurde das MDA für mobile Endgeräte (Tablets, Smartphones) in digitaler Form als MDA-App weiterentwickelt [32] und bereits erfolgreich im Rahmen einer Studie angewandt. Dabei stand neben der Anwendbarkeit der App auch die Durchführung und Anwendung am Patienten durch Pflegekräfte im Vordergrund. Es zeigte sich, dass die Anwendung des digitalen MDA weniger fehleranfällig ist. Weiterhin wurde die digitale Form des MDA durch die Pflegekräfte in der Anwendung klar bevorzugt.

### Kaufunktionstest nach Huggare und Skindhöj

Der Kaufunktionstest nach Huggare und Skindhöj [10] basiert auf synthetischen Testgummis aus Carnaubawachs und Bariumsulfat, die einen Farbbinder enthalten. Der Proband wird gebeten, 4 Testgummis 10-mal zu kauen. Im Anschluss wird das zerkaute Testmaterial mit einer Erythrosinlösung versetzt. In Abhängigkeit von der Gesamtgröße der Oberfläche der zerkauten Partikel wird mehr oder weniger Farbstoff an die Partikel gebunden. Es besteht ein linearer Zusammenhang zwischen der Größe der Oberfläche und der Farbaufnahme durch die Partikel. Nach 30 min wird die Lösung abfiltriert und das Delta der Absorption gegenüber einer Standardlösung mit einem Spektralphotometer gemessen. Auf diese Weise lassen sich die Größe der freien Oberfläche der Testpartikel und damit das Ausmaß der Zerkleinerung des Testbolus bestimmen. Damit handelt es sich bei diesem Test prinzipiell ebenfalls um einen Zerkleinerungstest. Eine direkte Handlungsempfehlung geben die Autoren nicht.

### Kaufunktionstest nach Sato et al.

Sato et al. [27] beschreiben einen Kaufunktionstest, bei welchem aus 2 Farben (rot/grün) zusammengesetzte „Paraffin-wax“-Würfel von den Probanden gekaut werden. Dabei verfolgten die Autoren vorrangig das Ziel, die Nutzbarkeit der von ihnen beschriebenen Methode als Durchmischungstest zu untersuchen. Sie verwendeten daher eine zufällig ausgewählte Anzahl von Kauzyklen (5–50) und untersuchten lediglich an einer älteren Probandin die Reproduzierbarkeit der Ergebnisse von 5 Versuchen mit jeweils 10 Kauzyklen. Die Auswertung erfolgt mithilfe einer digitalen Bildanalyse, die prinzipiell mit der von Schimmel [29] beschrieben Methode vergleichbar ist. Eine direkte Handlungsempfehlung wird nicht gegeben; es werden lediglich 3 Kaueffizienzklassen vorgeschlagen.

### Kaufunktionstest nach Nakasima et al.

Beim Kaufunktionstest nach Nakasima et al. [23] wird dem Probanden eine Gummikapsel, gefüllt mit 730 mg Testgranulat, gegeben, welche aus einer Mischung verschiedener Bestandteile (u. a. Laktose, Zellulose, Maisstärke, Erythrosin) von 730 bis 740 Granulatteilchen besteht. Mit diesem Test wird primär die Kauleistung bestimmt. Die Testkapsel soll 15-mal vom Probanden gekaut werden. Während des Kauvorgangs wird das Testgranulat aufgebrochen, und rote Farbpigmente treten in die Kapsel aus. Nach dem Kauvorgang wird die Kapsel aufgeschnitten, das Testgranulat mit 20 ml destilliertem Wasser vermischt und 30 s verrührt. Dadurch wird unzerkleinertes Testgranulat vom zerkleinerten getrennt. Das zerkleinerte Granulat wird eluiert und der Erythrosingehalt der erhaltenen Lösung (Stärke der Verfärbung) mithilfe eines Spektralphotometers ermittelt. Es wird keine Handlungsempfehlung gegeben.

## Ausblick

Zusätzlich wäre auch eine Anwendung von Kaufunktionstests im Rahmen der aufsuchenden Betreuung denkbar. Um mögliche zahnärztliche Versorgungsengpässe in Bezug auf immobile Patientinnen und Patienten zu lösen, gibt es die Überlegungen, zahnmedizinische Fachangestellte (ZFA) mit mindestens dreijähriger Berufserfahrung in Anlehnung an allgemeinärztliche Konzepte (z. B. nichtärztliche Praxisassistenz [NäPa] in Mecklenburg-Vorpommern) speziell im Bereich der aufsuchenden Betreuung zu schulen. Hier könnte die Durchführung eines Kaufunktionstests der betreuenden Zahnärztin/dem betreuenden Zahnarzt zusätzliche Informationen geben.

Damit geriatrischen Assessments in der Zahnmedizin bekannt sind, gilt es diese bereits in die Ausbildung der Studierenden zu integrieren. Bisher gab es in Deutschland keine verpflichtend etablierten Lehrveranstaltungen im Fachgebiet der Gerodontologie, im Gegensatz zur Schweiz, wo das Fach bereits seit vielen Jahren mit einem festen Vorlesungsthemenkatalog und praktischen Kursen im zahnmedizinischen Studium fest verankert und obligat ist. Die neue Approbationsordnung für Zahnärzte (ZApprO) bietet nun die Möglichkeit, auch in Deutschland durch das Querschnittsfach „Medizin und Zahnmedizin des Alterns und des alten Menschen“ Lehrinhalte der Seniorenzahnmedizin zu vermitteln.

Vor allem sollte jedoch bei der Entwicklung und Durchführung zu Assessmentinstrumenten die Anwendung im Pflegealltag überprüft werden, denn nur, wenn sich diese Instrumente in den Alltag integrieren lassen, ist von einer regelmäßigen Anwendung durch Pflegekräfte oder Angehörige auszugehen.

## Fazit für die tägliche Praxis

- Durch die Zunahme der Lebenserwartung und den damit verbundenen Anstieg der Pflegebedürftigen werden Angehörige und Pflegepersonal mehr und mehr vor neue Herausforderungen in Bezug auf die Mundgesundheit der Pflegebedürftigen gestellt.
- Zur Detektion des Mundgesundheitszustandes sowie zur Feststellung, ob ein Patient zahnärztlich behandelt werden sollte, haben sich Assessmentinstrumente in Form von Kaufunktionstests etabliert.
- Kautests ermöglichen auch medizinischen Laien, Hinweise darauf zu erhalten, ob ein Termin in einer Zahnarztpraxis vereinbart werden sollte oder ein zahnärztliches Konsil notwendig ist.
- Unabhängig von den Ergebnissen der einzelnen Kaufunktionstests muss ein Patient, der Schmerzen beklagt, in jedem Fall einer zahnärztlichen Untersuchung und Betreuung zugeführt werden.
- Kaufunktionstests sind nicht geeignet, regelmäßige (jährliche) zahnärztliche Kontrolluntersuchungen zu ersetzen.
